# Meta-analysis of studies on biochemical marker tests for the diagnosis of premature rupture of membranes: comparison of performance indexes

**DOI:** 10.1186/1471-2393-14-183

**Published:** 2014-05-31

**Authors:** Montse Palacio, Maritta Kühnert, Richard Berger, Cindy L Larios, Louis Marcellin

**Affiliations:** 1 BCNatal - Barcelona Center for Maternal-Fetal and Neonatal Medicine (Hospital Clínic and Hospital Sant Joan de Deu), IDIBAPS, University of Barcelona and CIBERER Barcelona, Spain, Sabino de Arana 1, Barcelona 08028, Spain; 2 University Medical Centre, Hospital for Obstetrics and Perinatal Medicine, University of Marburg, Marburg, Germany; 3 Marienhaus Klinikum St. Elisabeth, Neuwied, University of Mainz, Mainz, Germany; 4 Medical Department, Clever Instruments S.L, Barcelona, Spain; 5 Université Paris Descartes, Maternité Port Royal, Hospital Cochin, Paris, France

**Keywords:** IGFBP-1, PAMG-1, PROM, Rapid test

## Abstract

**Background:**

Premature rupture of the membranes (PROM) is most commonly diagnosed using physical examination; however, accurate decision making in ambiguous cases is a major challenge in current obstetric practice. As this may influence a woman’s subsequent management, a number of tests designed to assist with confirming a diagnosis of PROM are commercially available. This study sought to evaluate the published data for the accuracy of two amniotic fluid-specific biomarker tests for PROM: insulin-like growth factor binding protein-1 (IGFBP-1 – Actim® PROM) and placental alpha microglobulin-1 (PAMG-1 – AmniSure®).

**Methods:**

Main analysis included all PubMed referenced studies related to Actim® PROM and AmniSure® with available data to extract performance rates. To compare accuracy, a comparison of pooled indexes of both rapid tests was performed. Studies in which both tests were used in the same clinical population were also analysed. Membrane status, whether it was known or a suspected rupture, and inclusion or not of women with bleeding, were considered.

**Results:**

All the available studies published in PubMed up to April 2013 were reviewed. Data were retrieved from 17 studies; 10 for Actim® PROM (n = 1066), four for AmniSure® (n = 1081) and three studies in which both biomarker tests were compared directly. The pooled analysis found that the specificity and positive predictive value were significantly higher for AmniSure® compared with Actim® PROM. However, when 762 and 1385 women with known or suspected rupture of membranes, respectively, were evaluated, AmniSure® only remained significantly superior in the latter group. Furthermore, when the two tests were compared directly in the same study no statistically significant differences were observed. Remarkably, women with a history or evidence of bleeding were excluded in all four studies for AmniSure®, in two Actim® PROM studies and in two of the three studies reporting on both tests.

**Conclusions:**

No differences were observed in the performance of the two tests in studies where they were used under the same clinical conditions or in women with known membrane status. Although AmniSure® performed better in suspected cases of PROM, this may need further analysis as exclusion of bleeding may not be representative of the real clinical presentation of women with suspected PROM.

## Background

Disruption of foetal membranes prior to the onset of labour, commonly known as premature rupture of membranes (PROM), is a frequent complication of pregnancy [1,2]. PROM occurs in 8 - 10% of all pregnancies [3] and pre-term PROM (PROM <37 weeks’ gestation) is associated with approximately a third of all premature births [1,2].

Often considered as an inert gestational sac, foetal membranes have a stratified structure with special biochemical characteristics that provide them with the ability to adapt to the expansion that occurs during pregnancy, resulting from increasing foetal size and amniotic fluid. Foetal membranes are composed of two layers, the amnion which faces the amniotic cavity and the chorion which faces the decidua [4]. Membrane integrity is essential to ensure normal term pregnancy. Evidence suggests that the mechanisms involved in the rupture of membranes include biochemical, immunologic and bacteriologic events. Currently, it is widely accepted that term or preterm rupture is associated with structural changes, caused by inflammatory processes induced by endocrine or infectious triggers [5,6].

The main complications and consequences of PROM are related to the gestational age at which it occurs, the latency until birth, concomitant infection of the gestational tissues which may impact both foetal and maternal outcomes, in addition to conditions specific to the foetus, such as oligohydramnios, cord compression, abruptio or cord prolapse [2]. The accurate diagnosis of PROM coupled with appropriate obstetric interventions, according to gestational age, are of key importance to limit the potential risk posed by these adverse maternal and foetal outcomes.

Without clear evidence of amniotic fluid loss observed by speculum examination, the diagnosis of PROM can be uncertain and complementary diagnostic tests are frequently needed. The diagnostic confirmation in ambiguous cases is a major challenge in current obstetric practice, because correct diagnosis is necessary in order to decide upon the most appropriate management and ultimately to reduce both maternal and foetal complications. The optimal test should be specific for amniotic fluid and not be affected by contamination from other corporal substances or vaginal medications. Multiple tests with varying performance, are available in order to assess the integrity of foetal membranes [7,8], including cytological, biochemical, or colorimetric and ultrasound techniques. Limitations of the accuracy of tests, e.g. poor specificity (i.e. a high proportion of false positives), may lead to unnecessary interventions such as hospitalisation, antibiotic therapy, application of corticosteroids [9,10] and even induction of labour [3,9,10]. In contrast, poor sensitivity (i.e. a high proportion of false negative results) may be reassuring and delay or deprive women of appropriate treatments [2], increasing the risk of potential maternal and foetal morbidity and mortality. Traditional bedside and non-invasive tests, such as the fern and nitrazine test, have a high rate of false-negative and false-positive results in cases where women have vaginal infections or the presence of semen, blood or topical antiseptics [1,3].

New non-invasive tests have been developed in the last 15–20 years, with a simple dipstick test format, based on the detection of specific proteins found in amniotic fluid and which combine high sensitivity rates with low false-positive results. There are a number of rapid immunoassay tests commercially available, of which the most commonly used are Actim® PROM (Medix Biochemica, Kauniainen, Finland), designed to detect insulin-like growth factor-binding protein-1 (IGFBP-1), and AmniSure® (Qiagen, Hilden, Germany) which detects the presence of placental alpha macroglobulin-1 (PAMG-1).

IGFBP-1 is an excreted protein synthesised in the decidual cells and foetal liver and detected in amniotic fluid throughout pregnancy [11-14]. Although serum concentration of IGFBP-1 increases with gestational age [12], it is found at considerably lower concentrations in maternal serum compared to amniotic fluid. This concentration difference is also described for PAMG-1 [14], although reported concentration data vary between publications [15]. Biomarker and rapid test characteristics are shown in Table 1. Samples for both tests are collected with a sterile polyester swab before vaginal examination and/or vaginal ultrasound. The sample is collected from vaginal fluid and extracted by placing the swab in a buffer containing a solvent, with the lower end of the strip submerged.

**Table 1:** Biomarker and rapid test characteristics

	**Biomarker**	**Biomarker concentrations in bodily fluids**		**Rapid test characteristics**	
		**Maternal blood**	**Amniotic fluid**	**Threshold**	**Time to obtain results**
Actim® PROM	IGFBP-1	29–300 μg/L [12,14]	10,500–350,000 μg/L [12,14]	>25 μg/L [12,14]	<5 minutes, if positive At 5 minutes, if negative
AmniSure®	PAMG-1	2.5–12.5 ng/ml*	2,000–25,000 ng/ml*	>5 ng/ml*	10 minutes after sampling False results after 15 minutes

*Unpublished values stated in the AmniSure® manufacturer’s instructions for use and information material

The aim of this study was to compare the available information on two of the most commonly used commercially available rapid tests for the diagnosis of PROM. This study sought not only to critically evaluate the published evidence on the use of IGFBP-1 (Actim® PROM) and PAMG-1 (AmniSure®) tests and make a comparison of their performance indices (sensitivity, specificity, positive predictive value [PPV] and negative predictive value [NPV]) for the diagnosis of PROM, but also to identify any variants that may influence the reported performance of both tests. These variants included the diagnosis status groups (known membrane status and suspected membrane rupture) at the time of inclusion of patients in the study and the inclusion/exclusion of women with evidence of bleeding. For this meta-analysis, pooled sensitivity and specificity rates were calculated based on the results of those studies which directly compared both tests in the same clinical setting. The results of this meta-analysis are of potential value to physicians to help them in their choice of rapid test to aid in the diagnosis of PROM.

## Methods

This analysis was conducted in accordance with PRISMA (Preferred Reporting Items for Systematic Reviews and Meta-Analyses) guidelines (see Additional file 1: completed PRISMA 2009 checklist).

A search of the PubMed database was conducted to identify all published studies, up to April 2013, relating to the rapid tests Actim® PROM and AmniSure®, without language restrictions and using a combination of the predefined search terms: PAMG-1 test, IGFBP-1 test, PAMG-1 PROM test, IGFBP-1 PROM test, placental alpha microglobulin-1 PROM test, insulin-like factor binding protein-1 PROM test, AmniSure® and Actim® PROM.

All abstracts, full texts and citations were reviewed to select the papers in which: a) the rapid tests were used as a tool to diagnose or complement diagnosis of a rupture of membranes in a clinical setting, where b) the confirmation on the final membrane status through a reference method was available in the paper and where c) the results of the test performance, through sensitivity and specificity or the raw number of positive and negative test results, were available. All articles which were not consistent with these criteria were excluded from this analysis.

Data extracted from each study included: year of publication, inclusion and exclusion criteria of the study (e.g. active bleeding), gestational age at test performance (range), number of women excluded and rationale for exclusion, reference method used to confirm PROM, condition of women at the beginning of the study (total women with suspected and confirmed or non-confirmed PROM), rapid test results and diagnosis (ruptured membranes or intact membranes) at final evaluation. When values of true positive (TP), false negative (FN), true negative (TN) and false positive (FP), were not explicitly reported, these were estimated based on sensitivity and specificity values and confidence intervals reported in the original publications.

To provide an estimation of the predictive performance of the tests, the sensitivity, specificity, PPV and NPV results for each study were calculated according to the Newcombe efficient-score method (corrected for continuity) [16], taking into account only the number of women with confirmed diagnosis of rupture according to the reference method in each study (*per protocol*, cases of suspected PROM without later confirmation of the diagnosis were not included in the final analysis). To further explore the results of this pooled analysis, a *post-hoc* comparison along with 95% Confidence Intervals (CIs) was also performed using the chi-square test, between each test result for subgroups whose membrane status was known and those who had a suspected membrane rupture, in order to explore reasons for potential differences. In this comparison, known membrane status refers to those women for whom membrane integrity status was clearly defined, i.e. women without any symptoms or suspicion of PROM and women who had an artificial rupture. Suspected membrane rupture refers to women whose membrane status was not known upon study entry and who were being evaluated for a suspected rupture. All probability values were 2-tailed and were corrected for multiple testing, and p ≤ 0.05 was considered statistically significant. All the statistical analyses were performed using Excel 2007 and SPSS 19.0 for Windows.

The results are presented for each test considering the pooled data and then stratified according to whether the membrane status was known (intact or ruptured) or PROM was clinically suspected.

## Results

**Figure 1:**
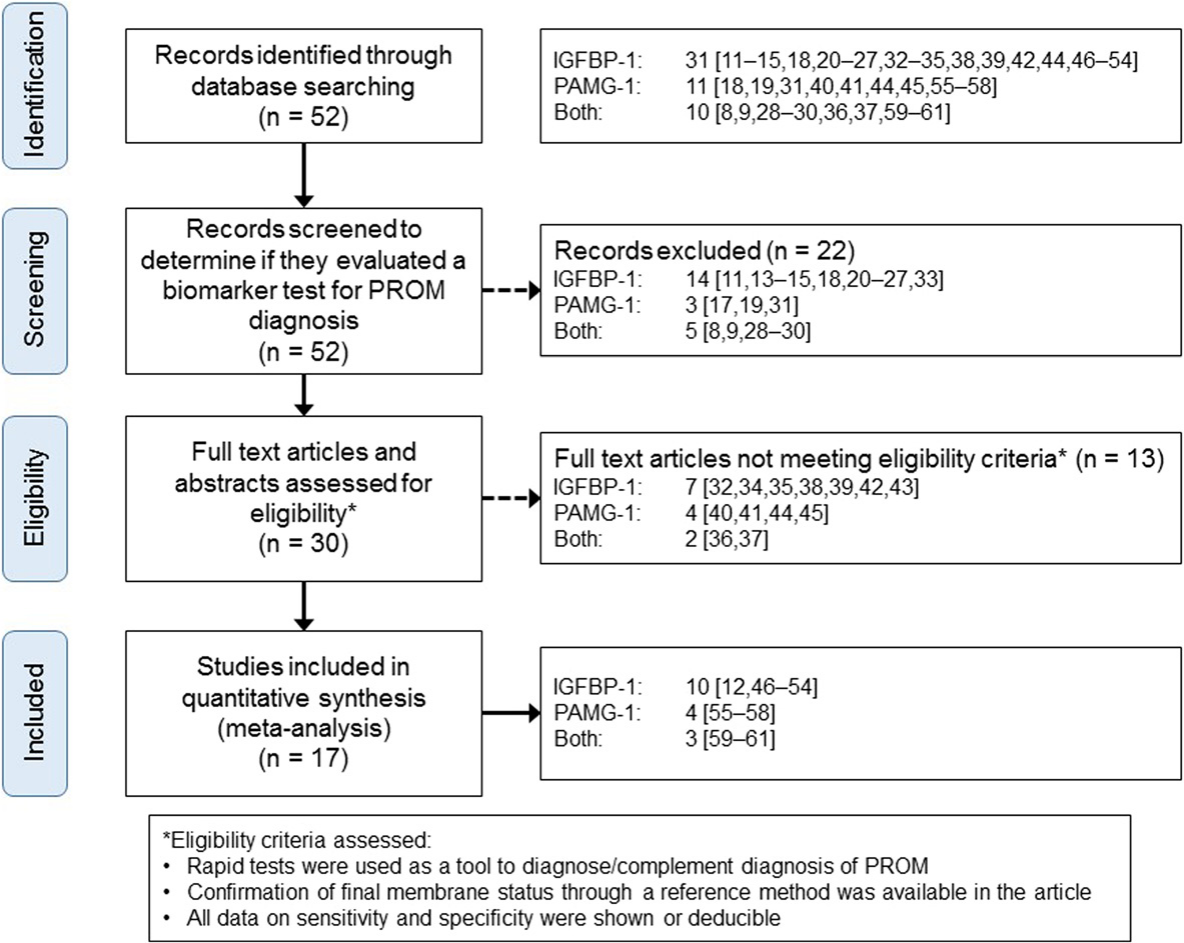
Flow chart of the selection process.

From an initial 125 identified manuscripts, all the retrieved titles and abstracts were screened to discard repeated articles, leading to a total of 52 evaluable papers: 31 papers relating to Actim® PROM, 11 papers relating to AmniSure®, and 10 referring to both biomarkers. After a detailed process of selection (Figure 1), 35 papers were excluded because: they did not evaluate the specific biomarkers as a rapid test for PROM diagnosis [14,17-19], they studied the concentration of the biomarkers through pregnancy [11,20-23] or after an amniocentesis [24], they were solely studies of biochemical processes [25,26], they were adjunct to a genetic study [27], they comprised guidelines [9], they were review articles [8,13,28,29], they were a meta-analysis [30] or letter/comments on other articles [15,31-33], and they related to the application of the biomarker in obstetrics [18,23]. In two cases, the full text version of the studies were not available for consultation [34,35]. The rest of excluded publications: did not evaluate the commercially available test in a daily clinical setting (i.e. they were *in vitro* studies [36-38], or presented test results mixed with other test modalities [39], they evaluated physicians’ confidence on PROM suspicion after the test [40], they presented incomplete data on sensitivity and specificity [41] or used an inadequate reference method to confirm PROM diagnosis [42,44]). One study on AmniSure® [45] had been retracted from publication due to inaccurate results, and thus it was also excluded from the analysis. Reasons for exclusion and a detailed flow chart of the selection process are presented in Figure 1.

Following this screening, data from a total of 17 selected publications were retrieved for analysis: 10 for Actim® PROM [12,46-54], four studies for AmniSure® [55-58], and three studies which evaluated both tests [59-61]. A complete description of the evaluated trials, including total samples and exclusion rationales is presented in Table 2. There were differences between the studies relating to the methods applied to confirm the diagnosis after the test had been performed (i.e. different concept of gold standard). Inclusion criteria for all studies were similar, except in two evaluating Actim® PROM [50,54], four studies evaluating AmniSure® [55-58], and two studies evaluating both tests [59,61], in which women with a history of bleeding or active bleeding at the time of evaluation were systematically excluded.

**Table 2:** Descriptive data of studies included in the meta-analysis

**Reference**	**GA range**	**N (ITT)**	**Women excluded from reference analysis**	**Rationale for exclusion from reference analysis**	**Exclusion of women with history or active bleeding**	**Reference method**	**N (PP)**	**Women excluded from meta-analysis**	**Rationale for exclusion of women from meta-analysis**
**Actim® PROM(IGFBP-1)**									
Rutanen 1996 [12]	15–37	311	0	--	No	For women with suspected ROM but equivocal diagnosis, ROM was assessed based on the interval between sampling and delivery.	130	181	Women with suspected ROM but inadequate reference method to confirm PROM.
Ragosch 1996 [50]	22-41	75	0	--	Yes	Clinical confirmation in cases of obvious PROM. Women with unconfirmed PROM underwent amniocentesis (if patient consented).	75	0	
Gaucherand 1997 [53]	19-41	100	0	--	No	Clinical course and similarity of the majority of the three tests: Actim® PROM, DAO and pH.	69	31	Women with suspected ROM and PROM confirmation absent.
Jain 1998 [49]	24-42	100	0	--	No	Pooling of liquid in the posterior fornix or seen leaking from the cervix.	100	0	--
Kubota 1998 [46]	15-41	48	0	--	No	At delivery and/or by observing the subsequent clinical course.	48		--
Darj 1998 [51]	25-42	174	0	--	No	Delivery within 48 hours. Method not available for women with suspected PROM.	75	99	Women with suspected ROM but inadequate reference method to confirm PROM.
Guibourdenche 1999 [48]	18-41	80	0	--	No	For women with suspected PROM, diagnosis was confirmed using detection of diamine oxidase in vaginal secretions (detected by semi-quantitative radio-enzymatic assay).	30	50	Women with suspected ROM and PROM confirmation absent.
Erdermoglu 2004 [47]	20-42	151	0	--	No	Speculum examination at inclusion. Later diagnosis in suspected women was associated with delivery within the next 7 days following the test.	71	80	Women with suspected ROM but PROM confirmation absent.
Akercan 2005 [54]	20-36	87	6	Lost on follow-up (4) and 2 who refused hospital admission.	Yes	Ongoing vaginal fluid leakage and/or ruptured amniotic membranes at first vaginal examination. Pooling of amniotic fluid in the posterior fornix.	45	36	Women with suspected ROM but inadequate reference method to confirm PROM.
Martinez de Tejada 2006 [52]	24-41	83	0	--	No	Presence of AF in the vagina or total absence of vaginal secretions, alkaline pH, positive fern test, oligohydramnios (AFI <5 cm), chorioamnionitis, absence or very little amount of AF leakage during labour and delivery.	83	0	--
Tagore 2010 [59]	17-37	100	6	Not specified on paper.	Yes	Three or more of: definite pooling of clear fluid during speculum examination, oligohydramnios on ultrasound, signs and symptoms of chorioamnionitis and preterm delivery within a week of presentation along with convincing history of leaking liquor.	94	0	--
Albayrak 2011 [61]	16-41	179	12	Lost on follow-up.	Yes	Speculum examination (clear fluid leakage) and two of: sonographic AFI, Fern, pH. Further dx was confirmed based on the duration of latency period, results of repeat speculum examinations, repeat ferning, nitrazine and strip tests and decrease of AFI, and clinical signs of foetal distress or chorioamnionitis.	167	0	--
Marcellin 2011 [60]	Not specified	80	1	Patient from the PROM group with placenta previa.	Not specified	Evident liquid outlet at vaginal examination.	79	0	--
**AmniSure®(PAMG-1)**									
Cousins 2005 [55]	15-42	203	0	--	Yes	Two of: visual pooling of AF, alkaline pH, positive Fern test.	203	0	--
Lee SE 2007 [56]	11-42	184	1	Lost on follow-up.	Yes	Leaking from the cervical OS on speculum examination or two of: visual pooling of fluid in the posterior fornix, positive nitrazine test or positive fern test.	183	0	--
Tagore 2010 [59]	17-37	100	0	--	Yes	Three or more of: definite pooling of clear fluid during speculum examination, oligohydramnios on ultrasound, signs and symptoms of chorioamnionitis and preterm delivery within a week of presentation along with convincing history of leaking liquor.	100	0	--
Marcellin 2011 [60]	Not specified	80	1	Patient from the PROM group with placenta previa.	Not specified	Evident liquid outlet at vaginal examination.	79	0	--
Albayrak 2011 [61]	16-41	179	12	Lost on follow-up.	Yes	Speculum examination (clear fluid leakage) and two of: sonographic AFI, Fern, pH. Further diagnosis was confirmed based on the duration of latency period, results of repeat speculum examinations, repeat ferning, nitrazine and strip tests and decrease of AFI, and clinical signs of foetal distress or chorioamnionitis.	167	0	--
Birkenmaier 2012 [57]	17-42	202	3	Excluded retrospectively due to incomplete medical records.	Yes	Two of: visual leaking or pooling of AF from the cervix on the speculum examination, positive nitrazine test or AFI <5 cm in the ultrasound examination. Definitive dx was diagnosed on review of medical records when there was documented evidence of intact or ruptured membranes with consecutive loss of fluid during delivery.	199	0	--
Abdelazim 2012 [58]	>37	150	0	--	Yes	History of sudden gush of water, pooling of AF, positive ferning pattern, positive nitrazine test and confirmed by visualisation of fluid passing from the cervical canal during sterile speculum examination.	150	0	--

AF: Amniotic fluid; AFI: Amniotic fluid index; GA: Gestational age in weeks; ITT: Intention to Treat; PP: Per Protocol; DAO: Diamine oxidase.

The pooled population consisted of 1066 pregnant women tested with IGFBP-1 and 1081 tested with PAMG-1. Prevalence of PROM as a final diagnosis was approximately 50% for both tests.

Performance indices calculated with the estimated pooled data of Actim® PROM (TP = 478, FN = 23, TN = 525, FP = 40) and AmniSure® (TP = 530, FN = 18, TN = 524, FP = 9) showed no statistical differences regarding sensitivity (Actim® PROM: 95.4% [95% CI = 93.1–97.0] *vs.* AmniSure®: 96.7% [95% CI = 94.8–98.0]; p = 0.352) and NPV (Actim® PROM: 95.8% [95% CI = 93.7–97.3] *vs*. AmniSure®: 96.7% [95% CI = 94.7–98.0]; p = 0.548). However, AmniSure® was associated with a higher specificity (98.3% [95% CI = 96.7–99.2]) and PPV (98.3 [95% CI = 96.7–99.2]) compared with Actim® PROM (specificity = 92.9% [95% CI = 90.4–94.8]; PPV = 92.3% [95% CI = 89.5–94.4]; both p < 0.001 *vs*. AmniSure®).

**Figure 2:**
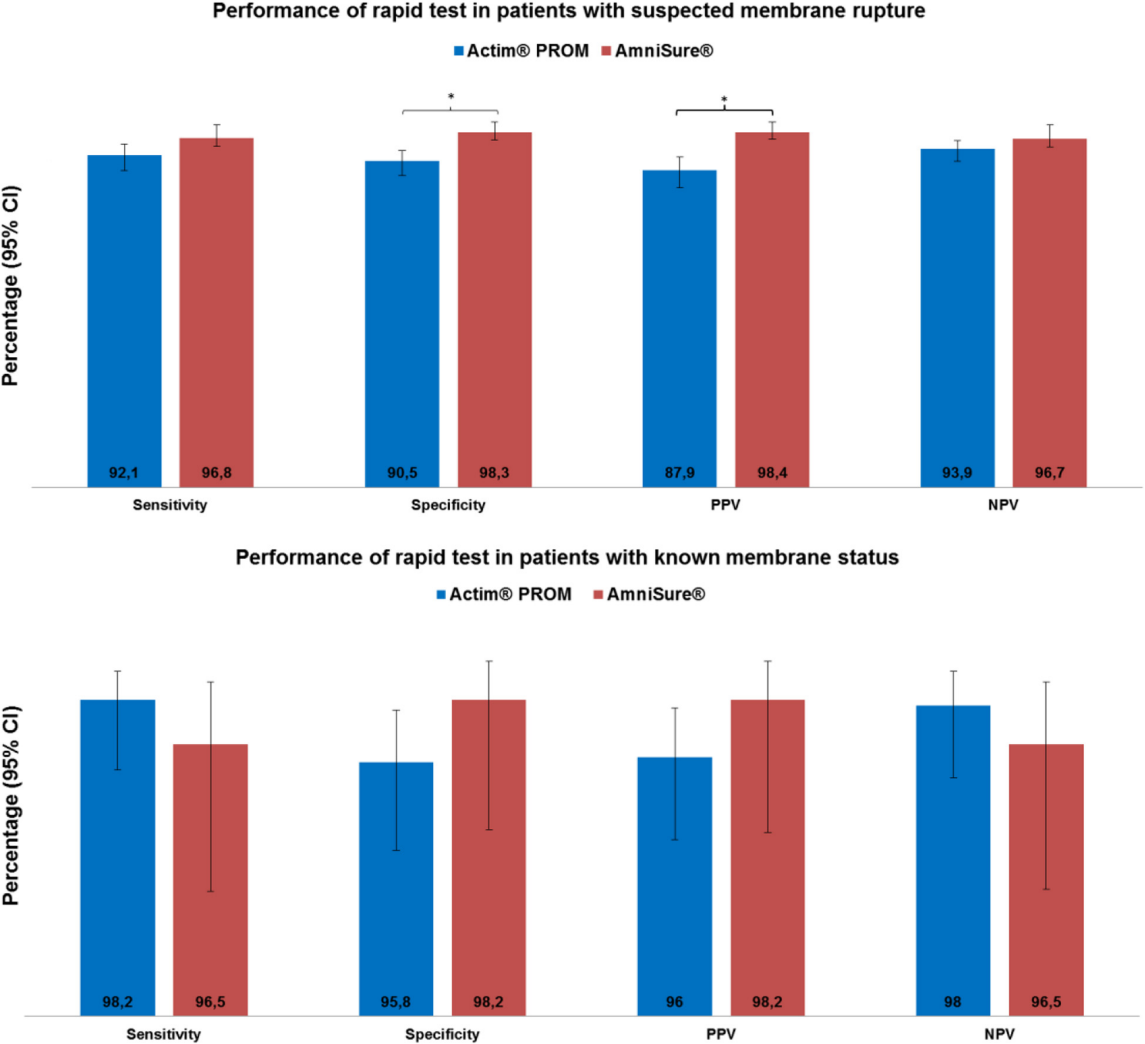
**Comparison of the performance indexes in known and suspected PROM populations.** *p<0.001. CI: 95% Confidence Interval; NPV: Negative Predictive Value; PPV: Positive Predictive Value.

Following the differences observed between the two tests in the pooled analysis, a *post-hoc* analysis of subgroups was undertaken to explore the potential reasons for these differences. Women included in the identified published studies were a mixed population and most studies included two types of patients. 1) Women with a confirmed membrane rupture or intact membranes; in these studies the women were used to evaluate the tests as true positive or true negatives, to show the efficacy of the tests in women with known membrane status. 2) Women who were suspected of having a membrane rupture; these represent the women who are relevant in the clinical utility of these tests and studies on these women evaluated the efficacy of the tests in the clinical setting. The overall population was stratified into women with known (Table 3) or suspected rupture of membranes (Table 4), where 762 and 1385 women, respectively, were evaluated. In this case, specificity and PPV only remained significantly higher for AmniSure**®** in the population where rupture of membranes was suspected. There were no differences between the two tests when they were compared in the group of women with known membrane status. A comparison of the performance indices in both populations is shown in Figure 2. Furthermore, in three studies, the two tests were compared directly in the same population. In these studies there was no statistically significant difference in any of the performance metrics of Actim® PROM compared with AmniSure® (Table 5).

**Table 3:** Summary of study results for women with known membrane status; women included in studies who had a confirmed membrane rupture or intact membranes (not suspected of PROM) upon entry to the study

**Study**	**N**	**TP**	**FN**	**TN**	**FP**	**Sensitivity% (CI)**	**Specificity% (CI)**	**PPV% (CI)**	**NPV% (CI)**
Marcellin 2011 [60]	79	39	1	38	1	97.5 (85.7–100)	97.4 (82.4–99.4)	97.5 (88.5–100)	97.4 (92.5–100)
Martinez de Tejada 2006 [52]	34	20	0	13	1	100 (79.9–100)	92.8 (64.2–99.6)	95.2 (74.1–99.7)	100 (71.7–100)
Akercan 2005 [54]	45	25	0	19	1	100 (83.4–100)	95.0 (73.1–99.7)	96.1 (78.4–99.8)	100 (79.1–100)
Erdermoglu 2004 [47]	71	35	1	34	1	97.2 (83.8–99.8)	97.1 (83.4–99.8)	97.2 (83.8–99.8)	97.1 (83.4–99,8)
Darj 1998 [51]	75	44	2	27	2	95.6 (84.0–99.2)	93.1 (75.8–98.8)	95.7 (84.0–99.2)	93.1 (75.8–98.8)
Gaucherand 1997 [53]	69	34	1	34	0	97.1 (83.4–99.8)	100 (87.4–99.8)	100 (87.4–100)	97.1 (83.4–99.8)
Guibourdenche 1999 [48]	30	15	0	14	1	100 (74.6–100)	93.3 (66.0–99.7)	93.8 (67.7–99.7)	100 (73.2–100)
Rutanen 1996 [12]	130	55	0	71	4	100 (91.9–100)	94.7 (86.2–98.3)	93.2 (82.7–97.8)	100 (93.6–100)
**Actim PROM® pooled**	**533**	**267**	**5**	**250**	**11**	**98.2 (95.5–99.3)**	**95.8 (92.4–97.8)**	**96.0 (92.8–97.9)**	**98.0 (95.2–99.3)**
Abdelazim 2012 [58]	150	73	2	74	1	97.3 (89.8–99.5)	98.7 (91.8–99.9)	98.6 (91.7–99.9)	97.4 (90.0–99.5)
Marcellin 2011 [60]	79	38	2	38	1	95.0 (82.4–99.4)	94.8 (79.3–98.0)	95.0 (84.7–100)	94.8 (87.9–100)
**AmniSure® pooled**	**229**	**111**	**4**	**112**	**2**	**96.5 (90.8–98.9)**	**98.2 (93.2–99.7)**	**98.2 (93.1–99.7)**	**96.5 (90.9–98.9)**

CI: Confidence Interval; TP: True Positives; FN: False Negatives; TN: True Negatives; FP: False Positives.

**Table 4:** Summary of study results for women with suspected membrane rupture; women with unknown membrane status upon entry to the study but who had a suspected membrane rupture

**Study**	**N**	**TP**	**FN**	**TN**	**FP**	**Sensitivity% (CI)**	**Specificity% (CI)**	**PPV% (CI)**	**NPV% (CI)**
Albayrak 2011 [61]	167	79	9	77	2	89.8 (81 0–94.9)	97.5 (90.3–99.6)	97.5 (90.5–99.6)	89.5 (80.6–94.8)
Tagore 2010 [59]	94	35	5	51	3	87.5 (72.4–95.3)	94.4 (83.7–98.6)	92.1 (77.5–97.9)	91.1 (79.6–96.7)
Martinez de Tejada 2006 [52]	49	19	3	20	7	86.4 (64.0–96.4)	74.1 (53.4–88.1)	73.1 (51.9–87.6)	87.0 (65.3–96.6)
Kubota 1998 [46]	48	18	1	27	2	94.7 (71.9–99.7)	93.1 (75.8–98.8)	90.0 (66.9–98.2)	96.4 (79.8–99.8)
Jain 1998 [49]	100	25	0	67	8	100 (83.4–100)	89.3 (79.5–94.9)	75.8 (57.4–88.3)	100 (93.2–100)
Ragosch 1996 [50]	75	35	0	33	7	100 (87.7–100)	82.5 (66.6–92.1)	83.3 (68.0–92.5)	100 (87.0–100)
**Actim® PROM pooled**	**533**	**211**	**18**	**275**	**29**	**92.1 (87.7–95.1)**	**90.5 (86.4–93.4)**	**87.9 (82.9–91.6)**	**93.9 (90.3–96.2)**
Birkenmaier 2012 [57]	199	51	3	143	2	94.4 (83.7–98.6)	98.6 (94.6–99.8)	96.2 (85.9–99.3)	97.9 (93.6–99.5)
Albayrak 2011 [61]	167	83	5	77	2	94.3 (86.6–97.9)	97.5 (90.3–99.6)	97.6 (91.0–99.6)	93.9 (85.7–97.7)
Tagore 2010 [59]	100	38	3	59	0	92.7 (79.0–98.1)	100 (92.4–100)	100 (88.6–100)	95.2 (85.6–98.7)
Lee 2007 [56]	183	157	2	21	3	98.7 (95.1–99.8)	87.5 (66.5–96.7)	98.1 (94.2–99.5)	91.3 (70.5–98.5)
Cousins 2005 [55]	203	90	1	112	0	98.9 (93.2–99.9)	100 (95.9–100)	100 (94.9–100)	99.1 (94.4–99.95)
**AmniSure® pooled**	**852**	**419**	**14**	**412**	**7**	**96.8 (94.5–98.1)**	**98.3 (96.4–99.3)**	**98.4 (96.5–99.3)**	**96.7 (94.4–98.1)**

CI: Confidence Interval; TP: True Positives; FN: False Negatives; TN: True Negatives; FP: False Positives.

**Table 5:** Performance in studies with side by side clinical comparison

	**Actim® PROM**	**AmniSure®**	**P**	**Actim® PROM**	**AmniSure®**	**P**
	**Sensitivity,% (95% CI)**			**Specificity,% (95% CI)**		
Marcellin 2011 [60] N = 80	97.5 (85.7–100)	95.0 (82.4–99.4)	NS	97.4 (82.4–99.4)	94.8 (79.3–98.0)	NS
Albayrak 2011 [61] N = 167	89.7 (81.0–94.9)	94.3 (86.6–97.9)	0.768	97.5 (90.3–99.6)	97.5 (90.3–99.6)	1.000
Tagore 2010 [59] N =100	87.5 (72.4–95.3)	92.68 (79.0–98.1)	0.480	94.44 (83.7–98.6)	100 (92.4–100)	0.248

NS: Differences not statistically significant at <0.05. CI: Confidence Interval.

## Discussions

Based on clinical evaluation, PROM can be equivocal in 10 to 20% of women consulting due to suspected loss of vaginal fluid [2,33]. Improved diagnostic methods, using biochemical markers specific for amniotic fluid, have been developed and extensively studied in the last few decades. These biomarkers are found at higher concentrations in amniotic fluid compared with vaginal fluid and thus provide a strong predictive value for the diagnosis of PROM. Multiple studies have shown the superiority of the new generation of tests, which have improved ease of sample processing and accuracy, compared with ‘classic’ tests [7,47,53].

The main finding of this analysis was the fact that the two tests evaluated (Actim PROM® and AmniSure®) performed equally when they were compared directly under the same clinical conditions and where women with known membranes status were tested. Considering the estimated pooled data, AmniSure® showed a higher specificity and PPV than Actim® PROM. As a result of these differences, the *post-hoc* analysis of subgroups was performed to evaluate separately women with known membrane status from those with suspected rupture of membranes, finding that a higher specificity and PPV of AmniSure® was only observed in samples from cases of suspected rupture of membranes (Figure 2).

These observed differences between the two tests could possibly be linked to the consideration of active or a past history of bleeding in test evaluations. Six of the seven AmniSure® studies [55-59,61], explicitly excluded women when there was evidence of active bleeding, or even a history of bleeding. Considering the importance of this exclusion, we found that eight studies in which women with bleeding were excluded (four for AmniSure®, two for Actim® PROM and two for both tests) comprise more than 90% of the available data relating to women tested using AmniSure®, but only approximately 20% of data relating to women tested with Actim® PROM. This exclusion is most likely due to the reported interference of blood with the test performance of AmniSure® (according to manufacturer’s recommendations), leading to false-positive results. This is unlike Actim® PROM, which is understood to be efficient in almost all cases, including women with some bleeding. This is due to a) the cut-off detection limit for IGFBP-1 in Actim® PROM is >25 μg/L in the extracted sample, which corresponds to a concentration of >400 μg/L in the sample taken from the woman, which is well above the level found in maternal blood (29–300 μg/L) [12] (Table 1) and b) a low affinity of the antibody used in Actim® PROM for the highly phosphorylated form of IGFBP-1 which is predominant in blood [25]. Thus blood contamination is highly unlikely to affect the test result of Actim® PROM. Altogether, these data provide supporting evidence that blood contamination may have limited impact on Actim® PROM’s performance [12,46-48]. The presence of blood, in varying degrees, is observed in up to 20% of PROM cases, it is particularly common during the pre-labour period due to cervical ripening [12,46,47] or in cases of placental implantation abnormalities (i.e. placentae previa).

The exclusion of women with bleeding can consequently provide unrepresentative performance values of a test for PROM and may impact upon test accuracy. Indeed, as the threshold of the AmniSure® test is very close to the lower limit described as a normal range in the maternal serum (Table 1), it could be hypothesized that traces of blood would have resulted in more false-positive tests, thus limiting the specificity, while this threshold is well above the levels found in maternal blood for the Actim® PROM test. Therefore, the presence of traces of blood should not impact on the test results using Actim® PROM. In a recently published meta-analysis that concluded a superiority of the AmniSure® test compared with the Actim® PROM test (Ramsauer *et al.*[62]), this exclusion of women with contaminating blood in their samples was not considered. Therefore, the results of this analysis should be interpreted with caution.

Another strength of the meta-analysis reported here is that it only included studies which met well-defined criteria. In the meta-analysis by Ramsauer *et al*. [62] comparing the two tests, the criteria for selection of the studies were in some cases conflicting with the described methodology: some of the published data available at that time were not included [58,60] and some evidence on AmniSure® results could not be verified because it was published only in abstract form and not available as full text [41].

It should also be noted that the study for AmniSure® with the largest sample size [55] was performed with a version of the test that is no longer commercially available. Although instructions for use only vary slightly from the currently available test (the diluent with the sample was applied to a slide instead of a test strip dipped directly into the diluent vial), it is not known whether this new test strip format has any influence on the efficacy of AmniSure® for the diagnosis of PROM. Of interest is the fact that the prevalence of the final diagnosis of PROM in the pooled data is approximately 50%, which depicts the true nature of the conflicting diagnosis of PROM being evaluated. This meta-analysis thus reflects the clinical situation experienced by physicians, in which women presenting with suspected PROM have a final confirmed diagnosis in approximately 50% of cases.

Our results, however, are not exempt from limitations, mainly related to the high complexity involved in the evaluation of the performance of diagnostic tests and the possibility of misleading published studies which are not available through Medline searches, in addition to the heterogeneity of design across studies. These factors were considered and lead us to perform subgroup analysis, which included those papers in which the final outcome was an interpretation of the performance index presented by the authors of each publication.

Particularly when tests are evaluated in the clinical setting, when PROM is suspected, the specific characteristics of each test, the selection of the women and the reference method used to confirm the diagnosis may contribute to inconsistencies. This is due to the fact that in most of the studies available for consultation, the reference method was not clearly stated or was heterogeneous (included a composite reference method, which combined the results of several available tests [12]).

A number of statistical methods have been proposed to estimate the performance of tests in the absence of a single accepted reference standard [13,14]. The importance of the diagnostic criteria for assessment of the tests performance is particularly highlighted in the group of suspected cases, where the sensitivity and specificity rates vary strongly throughout the studies. These findings suggest that the women had heterogeneous clinical characteristics and were managed according to different protocols during the studies, i.e., regarding reference methods to confirm PROM. In contrast, prevalence rates as well as accuracy characteristics such as sensitivity, specificity, PPV and NPV from the analysed data of Actim® PROM and AmniSure® studies are reasonably homogeneous. Despite a higher number of published studies for Actim® PROM, the total number of women included in both rapid test studies is comparable.

Overall, this analysis shows that accuracy of Actim® PROM and AmniSure® for the detection of PROM are comparable if used in the same clinical population [59-61]. Although there are significant differences in the test performance in women with suspected membrane rupture, one should be cautious to conclude from this meta-analysis that under clinical conditions either test is superior in diagnosing PROM, as women with bleeding were mostly excluded when testing one of the biomarkers.

## Conclusions

In this analysis, both tests appear equally useful for clinical use to aid in the diagnosis of PROM, as no differences were observed between the tests when compared side by side in the same study. The exclusion of women with bleeding from all but one of the AmniSure® studies may limit direct comparison of the studies evaluating these two biomarkers. As some degree of bleeding may be present in a significant number of women presenting with suspected PROM in the real clinical setting, further studies are necessary to consider the performance of AmniSure® in such conditions.

## Abbreviations

FN: False negative; FP: False positive; IGFBP-1: Insulin-like growth factor binding protein-1; PPV: Positive predictive value; PAMG-1: Placental alpha microglobulin-1; PROM: Premature rupture of the membranes; NPV: Negative predictive value; TN: True negative; TP: True positive.

## Competing interests

Montse Palacio has previously received honoraria from Alere for an oral presentation. Richard Berger has previously received honoraria from Alere for oral presentations and advisory board attendance. Maritta Kühnert has previously received honoraria from Alere for advisory board attendance. LM has no competing interest to declare. Cindy L. Larios is part of the Medical Department of Clever Instruments, Barcelona, Spain, which is an independent CRO.

## Authors’ contributions

MP participated in the interpretation of the meta-analysis data and the preparation of the manuscript. RB, MK and LM participated in the interpretation of the data and in the critical review and revision of the manuscript draft. CLL performed statistical analysis for the study and participated in the preparation of the manuscript. All authors read and approved the final manuscript.

## Pre-publication history

The pre-publication history for this paper can be accessed here:

http://www.biomedcentral.com/1471-2393/14/183/prepub

## Supplementary Material

Additional file 1 Prisma 2009 checklist. Click here for file
